# Lessons learned about primary weight maintenance and secondary weight maintenance: results from a qualitative study

**DOI:** 10.1186/s12889-015-1930-z

**Published:** 2015-06-24

**Authors:** Ann Reilly, Barbara Mawn, Davide Susta, Anthony Staines, Sarah Browne, Mary Rose Sweeney

**Affiliations:** NURISH, School of Nursing & Human Sciences, Dublin City University, Dublin, Ireland; University of Massachusetts, Lowell, Boston, USA; School of Health and Human Performance, Dublin City University, Dublin, Ireland

**Keywords:** Primary weight maintenance, Weight loss maintenance, Failure to lose weight

## Abstract

**Background:**

Obesity is now a worldwide problem and Ireland is no exception with approximately two thirds of the adult population now overweight or obese. A recent report has found that 53 % of Irish adults aged 50 years and over are classified as centrally obese and at substantially increased risk of metabolic complications. While most studies investigating weight maintenance have been conducted on those who have managed to lose weight and/or achieved weight loss maintenance (secondary weight maintainers), few studies have been undertaken to understand the attitudes, behaviours, motivations and strategies of those who maintain their weight within normal weight ranges over their lifetime, so called primary weight maintainers. This study aims to explore this issue through qualitative exploration of primary weight maintainers in an Irish University.

**Methods:**

Seven focus groups were conducted (including three single interviews) with 17 participants in total across three different groups, 1) primary weight maintainers, 2) secondary weight maintainers, and 3) those unable to sustain or achieve weight loss. The interviews were transcribed and thematic analysis was applied to interpret the findings.

**Results:**

After analyzing the participant’s interviews, planning and organization or lack of, emerged as themes across the three groups in varying degrees. Strategizing, perseverance and willpower were seen as integral to weight maintenance and weight loss in groups one and two, these were lacking in group three. Prioritizing exercise and perseverance in maintaining a high level of activity was evident in groups one and two and was lacking in group three. Motivational influences were equal across the groups however, group three found it difficult to turn this into action. Group one had behavioural control of calorie intake maintaining a balance between week and weekend eating. Group three found it difficult to control calorie intake and portion size. Self-image differed across the three groups with cognitive dissonance evident amongst those in group three.

**Conclusions:**

This study showed that there are many factors that influence primary weight maintenance. Considering that we live in a society that is predominantly sedentary, predominantly overweight and with poor food choice options facing us every day, fighting our way through to ensure healthy weight maintenance requires active, conscious efforts. The factors identified in this study which are important in healthy weight maintenance are all potentially modifiable with life-coach, nutrition, exercise and cognitive interventions particularly if peer support and a whole family approach are incorporated.

## Background

Among the developed countries of the world, the cause of obesity has been attributed to a complex web of behavioural, genetic, social, cultural, political, economic and environmental factors. Overweight and obesity are becoming more prevalent across the entire spectrum of socio-economic groupings. The World Health Organisation (WHO) estimates that approximately 1.9 billion adults aged 18 years and over worldwide (39 %) are overweight with 600 million (13 %) obese [1]. The impact of obesity is considerable on a personal and societal level. It places an individual at increased risk for heart disease, stroke, diabetes, sleep apnoea, musculoskeletal disorders, depression, psychological consequences and some cancers [2, 3].

Despite a plethora of interventions among various target populations globally, the trend to increased obesity continues across all ages. Approximately two thirds of the adult population in Ireland are now overweight or obese as well as 30 % of 9 year old girls and 22 % of 9 year old boys [4]. A recent report has found that 53 % of Irish adults aged 50 and over are classified as centrally obese and at substantially increased risk of metabolic complications [5]. Ireland now ranks as one of the most overweight/obese nations in the European Union [6]. To address this concern, the Minister of Health and Children appointed a National Task Force in 2005 to review the obesity trends in Ireland and to make recommendations for policy change [7], and has more recently (2011) established a special action group on obesity [8] to take action across a range of areas. While there has been little previous research on the population who successfully maintain their weight in the “normal ranges” (Body Mass Index (BMI) from 18.5-24.9) otherwise known as primary weight maintainers, much research has focused on secondary weight maintenance, that is achieving and maintaining weight loss. In their analysis of weight loss maintainers and weight loss re-gainers, Kayman et al [9] conducted telephone questionnaires to identify differences between the groups. Kayman et al’s findings suggest that those who maintain an average weight (control group) were more likely to be conscious of the type of food consumed and the quantity. The control group also used more problem focused, than emotion focused coping in response to problem situations, and sought out social supports often. Considering the current statistic that 24 % of adults between the ages of 18 to 64 are obese and 37 % are overweight, 39 % therefore fall into the normal weight range (BMI 18.5-24.9) [10]. We believe there is much to learn through engaging with this group of individuals who fall into the normal weight ranges with regards to their attitudes, knowledge, health beliefs, behaviours, cognitive processes, strategies and motivations with respect to diet, exercise and lifestyle. A deeper understanding of these factors could lead to the development of recommendations and even interventions which could promote healthy lifestyles and thus reduce or indeed avoid overweight and obesity. It has also been suggested that health promotion programs need to incorporate an “anytime, anyplace” program delivery model to address access barriers [11].

Targeting the factors that play a role in weight gain and/or dysfunctional eating may be important in managing weight loss and in weight maintenance [11]. In preceding reviews we notice the profile of “the successful weight loss maintainer” has been defined. It includes the following behaviours and cognitive abilities: active lifestyle and muscular activity in leisure time; effective self-regulation in terms of self-monitoring of eating habits, including reducing food intake, and healthy food awareness; self-sufficient, flexible control over eating behaviours; avoiding emotional instability; and effective stress management [12, 13]. The purpose of this study was to examine the behaviours, strategies and attitudes associated with primary weight maintenance, secondary weightmaintenance and weight gain amongst staff at an Irish University. We also sought to identify psychological and socio-cultural factors involved. Focus groups and in-depth personalised interviews were conducted with three groups of people.

## Methods

### Study design

This study sought to identify factors that influence primary weight maintenance amongst staff at an Irish University using a qualitative descriptive design. The focus of this qualitative study was to explore the manifest meaning of the data focusing on the everyday language of the participants. This study included staff working in an Irish University, aged above 18 years of age, employed by the University in any department or sector across the University. This group of participants comprised of a purposive sample that would form the basis for a much larger population study to be conducted in the future. Seven focus groups were conducted in total which included three single interviews amongst group three. Focus groups were chosen to encourage discussion amongst participants in an informal and more intimate setting.

### Sample

Participants were recruited using purposive sampling. A purposive sample is one that allows for participants to be purposively sought out and selected based on the knowledge of a population and the purpose of the study. Although not representative of the entire population, purposive sampling allows for the rich data gathered to test out or generate explanatory frameworks [14]. Quota sampling was then applied to allow the population under study to be divided into sub-groups fitting the three different groups related to weight control. There were seven focus groups in total including focus group one: two groups of staff who actively maintained their weight within a healthy range (Body Mass Index (BMI) 18.5-24.9) over their lifetime (n = 4 and 3), focus group two: one group of staff who had lost over 14 lbs (one stone) in weight and managed to maintain it for over one year (n = 4) and focus group three: four groups of staff whose BMI (24.9) or weight was considered overweight/obese who had tried repeatedly but had not lost weight in the past year (n = 3, 1, 1 and 1). Within group three individual interviews were offered due to the sensitive nature of disclosing information within a group that may cause discomfort or stress, and three such interviews were conducted within this group. There were 17 participants in total, five male (n = 5) and 12 female (n = 12). Participant’s ages ranged between 32 and 60 years of age with an average of 46 years of age. All participants were staff currently employed at an Irish University located in a major urban area. Staff across all sectors and departments (Support, Academic, Administrative and Technical) in an Irish University were invited to take part in the study. A flyer indicating the purpose of the study was designed by the interviewer and emailed to all sectors and departments using the internal email system ensuring all staff across the University would receive the information. Responses from participants who wished to take part in the study were collated and responded to by the interviewer. Participants were advised of the criteria for the three focus groups and were requested to self-select, based on their weight development, the group they fell into. Focus group discussions were decided based on the number of respondents who volunteered and the group sizes were deemed as appropriate. Interviews were then arranged between respective groups. Those who were not over 18, employed by or affiliated with the Irish University were excluded. Participant characteristics are detailed below in Table 1.

**Table 1:** Participant characteristics

Marital status	
Single	4
Married	11
Separated	1
Divorced	1
**Education**	
Inter/Leaving cert or equivalent	1
Diploma/Certificate	2
Primary Degree	3
Post Graduate	7
Higher Degree	4
**Income** '000	
20-39	1
40-59	6
60-79	3
80-99	4
100+	3
**Self-reported weight status**	
Normal weight	7
Overweight/Obese	6
Secondary Weight Maintainers	4

### Protocol

Ethical approval was granted by the DCU Research Ethics Committee. Participant’s responses to the flyer indicated their interest in the study and allowed for discussion between the participants and the interviewer, via email, regarding more detailed information on eligibility, which focus group they reported themselves as fitting into, whether participants preferred group or single interviews and availability for interview. Once agreement was reached and quota was filled, mutual appointments were agreed amongst each group. All focus groups including single interviews took part in the University itself in pre-booked rooms that could seat up to 15 people. On arrival participants were handed a questionnaire and a consent form. The purpose of the questionnaire was to obtain demographic information of participants. All participants were informed verbally prior to the interview that consent was voluntary and response to any questions that were posed was at the discretion of the participant. Participants were also advised if any discussion caused upset or discomfort they were free to leave the room at any stage or terminate the interview. A confidential counselling service was available to all participants if required. Participants were assured of anonymity in relation to their responses advising all transcripts after being transcribed by the interviewer would be held only by the principle researcher and would not be disclosed to others outside the research team. On completion of the transcription and analysis of the interviews, all transcripts would be deleted. Questionnaires and consent forms would be kept in a secure locked cabinet by the principle investigator. There were no unexpected occurrences during any of the interviews and all participants remained for the duration of the interviews. No incentives were provided for participation in this study. The interview guide was developed by the research team and questions are included in Appendix A. Each focus group and interviews took approximately one hour.

The focus groups and interviews were conducted by a trained interviewer, recorded and notes were taken by the assistant moderator. The assistant moderator noted specific quotes, included seating arrangements of participants, non-verbal clues and key points and themes that emerged. Each focus group and interviews were then transcribed verbatim by the interviewer, the transcripts were relayed back to the study participants to see if they reflected their intended meaning. In all cases they agreed that the transcripts had captured the essence of the conversations held.

### Analysis

Thematic analysis was used to analyse the data and comparisons were made across the three different groups [15, 16]. Thematic analysis is concerned with describing shared themes that occur across the data discussed by participants. Transcripts were read and re-read by the research team. Preliminary theme analysis were shared amongst the researchers for comparisons of identified themes. Following a review of the draft manuscript, refinement of themes occurred through a rigorous process of data familiarisation with final themes emerging in the manuscript following verbal agreement amongst the researchers. Following this process, themes were further refined which resulted in splitting themes into sub-themes to further reflect their meaning.

## Results

Within the three groups some interesting differences emerged in terms of attitudes, behaviours, strategies, resilience, emotional control, levels of family/social support and will power. The one thing which was consistent between the three groups was the desire or motivation to be slim, however only those in groups one and two had been able to fulfil this desire/motivation.

In our primary weight maintainers group weight maintenance was cited as an “absolute priority” in their lives driven by vanity and health in equal amounts. The size of their waist band was the main monitoring device for weight control. In other words if they felt the waist band on clothing getting tighter this signalled a time for action, prompted them to lower their calorie intake and increase their exercise regime for a few weeks until they were back fitting comfortably into their waist bands again. They would never allow themselves to buy a bigger clothes size. Neither fad diets, nor weighing scales were a feature in this group. They were passionate about food and seemed to have a very good relationship with food. Being slim was an integral part of their lives, and was driven by vanity, health and wanting to be a good parental role model. Willpower, balance, regulation, good emotional control, and resilience were strong themes emerging within this group. Organisation and planning were key to success. Food shopping had to be done regularly ensuring well stored cupboards, fridges and freezers. At home and at work almost all meals were planned in advance. Dining out was kept to an occasional treat rather than a routine. A pattern of strict dietary intake during week days emerged with more relaxed patterns of eating allowed at weekends with an acknowledgement that “treats” were necessary. Calories from alcohol were low with intakes during the week typically at zero with some intake allowed at weekends. Moderate and regular exercise featured strongly both for weight maintenance but also for the feel good factor and mental health. There was a good awareness of portion sizes and nutrient contents across the range of foodstuffs. Low fat foods did not feature amongst this group. Good family supports seemed to be an important element in facilitating these factors to come together.

In the group who had managed to sustain weight loss (group two) the participants identified a personalised approach that had worked for them and felt strongly regarding a “no one fit for all approach”. They spoke of having to find their own route that fitted with their particular lifestyle which illustrates the need for personalised support and advice tailored to the individual. Exercise had increased in frequency and intensity and portions sizes, planning and organisation had improved. In some cases support groups such as weight watchers had worked while others had make the transition alone. Family and peers at work were identified as being important in motivating and encouraging change and hence success. In this group there was an acknowledgement that the “battle was on-going” that there was pain involved and that a lifelong effort would be required. There would be no reverting to old habits. Reasons cited for being able to make the change included also vanity and health/health scares as well as landmark events such as getting married and having children. Perseverance was cited as key to success.

In group three there was an air of despondency with weight loss being almost a pre-occupation which never materialised. Most worried about their health long term but still could not change their behaviours. Vanity featured here also with the desire to fit into nicer clothes expressed. The cost implication of larger clothes was also alluded to. Some described self-loathing and low self esteem. Fluctuations in weight status were a lifelong feature. Disarray featured strongly in their lives as a whole and not just in relation to food. Lives were described as unstructured and lacking routine. Exercise was ad hoc and not sustained. Alcohol intakes were higher and seemed to be used as a comfort during times of despondency. Family support structures around childrearing was notably absent in this group making it harder to get time out to do shopping, prepare meals ahead or get space to exercise. Secret and guilt eating were a feature. Fad diets featured strongly with only short term weight loss achieved. Support groups such as weight watchers and slimming world featured strongly. Only one participant had ever consulted a dietician. Planning around food was absent, this seemed to result in this group skipping meals and eating later in the day and hence being more hungry and therefore consuming more than they would have done if they had planned ahead and had a meal to hand when they needed it. Skipping breakfast was also a feature. Participants reported binge eating and secret eating. Lack of will power and impulsivity were identified as problematic issues. Everybody in this group acknowledged that they eat too much, and that they didn’t exercise enough so a knowledge deficit does not seem to be a major issue. Critical time points when significant weight gain had occurred included pregnancy, marriage and times of emotional stress such as moving house, jobs and relationship breakups. The notion of rowing-back evident in group one did not feature in any sense in group three and there was a sense of their weight being totally out of control, as if there was nothing they could do about it. One subject likened it as to addiction similar to alcoholism. The idea of balance between calories consumed and expended did not emerge in any of the participants in group three. While all participants in this group cited weight loss as extremely important and desirable it was not prioritised in reality, nor in practice in their lives.

These results are discussed in more details in the next section under the main categories which emerged from the data.

### Theme: planning/organization

#### Sub-theme: being prepared

Being prepared and organised in relation to food and exercise was cited as important by participants in group one. Significant time was devoted to preparing lunches, having the fridge full, preparing dinners and having the gym bag packed and in the car. Planning was described by group one as ‘essential’. Improved planning, organization and dietary changes in order to encourage and maintain weight loss were reported by group two. Increased planning and better organisation included bringing in lunch, better use of freezer, However, for group three there was little or no planning around meals and meal times with other family members were sporadic.

*“I know I should plan and I want to plan but I never actually seem to succeed in tying it down”*

The lack of planning and organization around food often lead to skipped meals and hunger reaching levels which resulted in double portions being consumed at the next sitting, or grabbing take away food or ready to eat meals on the way home in the car.

*“..... if I haven’t planned which with the kids like they’re 8 and 10 and it’s mam this ma that, there’s hurling here and GAA there, and I’ve football there, and I’m dancing here, and I’m swimming there, and I’m everywhere. I’m working and I’m doing their homework and it’s all that tumbling down on top of you and you see you just grab whatever”*

### Theme: strategizing

#### Sub-theme: implementing techniques

Quick fix strategies were implemented by group one if they felt they had put on a small amount of weight by cutting back on treats and increasing exercise.

*“I decide OK cut back on the ice cream, the chocolate and the biscuits and maybe exercise a bit more”*

Group two also cited several strategies that were put in place to encourage and promote weight loss. Strategies implemented by participants included: calorie counting applications on phones, checking portion sizes on a digital scales.

*“and the only thing I really did was use an app on my iPhone, a calorie counter and I found it absolutely fantastic because it’s very easy to track what you eat”*

Others spoke of reducing portion sizes, reducing meats, increasing fish, veg, fruit and brown foods, reducing alcohol intake and keeping an eye on weight after socializing in order to ‘row back’ quickly. The aspect of strategizing was not seen in group three.

New behaviours such as steering clear of certain aisles in supermarkets was mentioned by those in group two such as and realizing that the majority of food in supermarkets are not needed. One respondent spoke of now avoiding the majority of the aisles in a supermarket

*‘I suddenly realized that 85 % of the stuff on the supermarket shelves you don’t actually need so I don’t go there’*

### Theme: perseverance

#### Sub-theme: determination

Participants in group one discussed how healthy food choices are often automatic and poor food choices are not selected routinely. For some rules are put in place regarding food choices with fish and nuts high on the list of must have foods and foods high in fat kept to a minimum. Picking the healthy dish would be because of choice rather than because it was expected.

Participant’s ability to persevere with weight loss efforts in group two was referred to as a lifelong struggle and one that was painful at times. Poor eating habits were replaced with better choices and alcohol consumption reduced. Development of discipline and determination were cited as necessary leading to improved confidence.

*“....determination, confidence and things become easier. Not sure why it’s easier, maybe because clothes start to fit better. It’s not a drama going out. Life is easier without the weight”*

Although ‘set-backs’ were experienced amongst those in group two, there was a recognition of continuous, conscious effort was required to sustain change.

For some participants in group three however, there was a sense of being stuck and an inability to control actions around food intake. Comparisons were drawn with alcoholism for one participant, with secret and binge eating a common theme that occurred throughout the group.

*“In a way I associate this almost similar to alcoholism it’s em.. sure now I don’t know but I would guess like an alcoholic in some part of their brains they know they shouldn’t be doing that or whatever somebody’s addicted to drugs it’s a another addiction as far as I’m concerned in some ways”*

Some specifically described behaviours of hiding food packaging from partners to avoid detection of over eating or fear of recrimination for their food choices.

*“I’d eat a bar of chocolate in secret and then hide the wrapper down the side of the sofa”*

#### Sub-theme: willpower

Willpower was strong in both groups one and two that included refusing food offered by others and weighing foods to ensure portion sizes are adhered. Being tired was cited as a reason for lack of willpower in group three, particularly in the evening times. For some not knowing why they could not restrict themselves was a source of frustration.

*“I buy a bar of chocolate - I don't know why I do it. I have these conversations in my head - can't seem to stop”*

Food is often seen as a reward, a treat for hard work or immediate gratification. Most participants advised that food was often consumed in excess due to lack of structure while poorer food choices were also made if a participant’s mood was low.

*‘At the end of the day the ‘willpower is gone to pot’.*

### Theme: prioritizing

#### Sub-theme: regular exercise

All participants in group one reported undertaking exercise in a sustained and systematic way often exercising three to four times per week. Early mornings and afternoon breaks were utilized frequently in order to fit exercise in during the day. Group one participants reported an interest in exercise, considering it as something that makes you feel good, is important for mental health and is challenging.

*“I would be interested in exercise, exercising itself and I suppose the competiveness side of it would attract so that would motivate me as well and then you’re challenging yourself to lose a bit of weight if you put it on”*

Regular sustained exercise was introduced by all participants in group two to assist weight loss. However it was noted as important by all participants that each must find their own way to keep an interest and ensure its sustainability.

*“finding your own way with regards to sport, for example a lot of people think oh I have to run or I have to gym no you have to find the thing that works your way”*

In groups one and two, exercise was preferred if it was undertaken as a normal part of the day *i.e.* cycling to work or as a social activity. However in group three, exercise was sporadic, ad hoc and was not carried out in a sustained way.

*“Never enjoyed exercise for its own sake - only as part of club or part of a normal day/social thing”*

### Theme: behavioural control

#### Sub-theme: balance

Weekday and weekend behaviours were implemented in group one who reported allowing themselves stricter guidelines during the week in order to have more relaxed rules at the weekend. Most participants in group one never allowed themselves to gain more than a small amount of weight before taking action to reverse it. This occurred in response to waist bands on clothes getting tighter and seeing themselves in photos looking heavier than they wanted to. This would lead to cutting out all junk foods and items such as butter, bread and cheese for a couple of weeks until they had lost the pounds gained. Weighing scales were not considered important for weight maintenance amongst most participants in group one who judged instead using clothing.

*“I judge by my clothes, my waist which is what usually happens to me is that I put on my waistband and I say oh now ease off on some of my favourite creams and butter and desserts”.*

Weekday and weekend behaviour also featured in group two with behaviours around food and alcohol considered good during the week allowing for slightly more relaxed rules at the weekend.

*“another very big behavioural change was you mentioned alcohol and I told myself I’m not going to drink alcohol from Sunday to Thursday................. there’s no way I’m not going to have a drink you know. I was conscious of it and I knew that I was going to have some alcohol Friday and Saturday but I would try to make up for it for the rest of the week”*

Keeping things in balance was important for weight loss according to group two also, who advised small ‘tweaks’ to the diet rather than big changes were undertaken by participants and it was agreed that this was necessary to keep things as normal as possible.

*“Once life stays the same you can do it – trying to change too much would make you miserable”*

In group three alcohol intakes were high and appeared to be used as a short term comfort. Trying to implement changes to immediately counteract excesses in diet or alcohol consumption did not feature in this group.

### Theme: motivational influences

#### Sub-theme: vanity

The motivations to maintain weight control in group one were diverse across the group with vanity considered a strong motivator.

*“Vanity is definitely something with me too I don’t like when I see photographs of myself and I think oh, that does motivate me”*

Vanity was also a motivational factor for participants in group two to make the changes necessary for weight loss often prompted by seeing themselves in photographs.

*“I saw a couple of photographs of myself in my around then in the middle late 40’s and I was unhappy with that and eh that’s my memory”*

Self-image featured in a very negative way amongst those in group three who had expressed feelings of disgust about how they looked and exhibited low self-esteem.

*“I feel disgusted when I look in the mirror”*

Being able to shop for and buy nice clothes was cited as a motivating factor for being slim amongst those in group one and two. Those in group three expressed dismay at not being able to fit into nice, inexpensive clothes and spoke of shopping and going out as a “*drama*”.

#### Sub-theme: major life events

Major life events such as getting married and having children were cited as strong motivational influences in group two which triggered weight loss

“*so I was getting married so I wanted to get a few lbs down so I actually lost the weight”*

#### Sub-theme: to maintain health

Health was considered on various grounds for all groups as a motivator. While for group one and two these factors often initiated change, for group three it remained a difficulty in turning this motivation into action.

*“I think what motivates me primarily is I want to be a healthy person em…… I don’t want to suffer any illnesses now or later and there’s so many illnesses that are related to inactivity”*

Other motivators amongst all groups included wanting to have energy and be able to play with their children, health scares/worries/family risk factors such as diabetes.

#### Sub-theme: support from family/colleagues/friends

Participants in group two particularly noted the psychological aspects of support as being key. Support was indicated by others undergoing weight loss or remarks made about appearance by colleagues/family and were regarded as having strong psychological aspects to weight loss success. Reading was also noted as an important support.

*“Being around others going through something similar is such a key thing”*

*“the support from colleagues and friends this is kind of you know when people see you let’s say every week or every two weeks and they say you look great, you lost some weight you know and that’s very motivating as well”*

Group three noted that lack of family support caused some difficulties for participants who were unable to make commitments to clubs or maintain schedules. For some lack of support made fitting meals around children difficult.

### Theme: perception

#### Sub-theme: recollection

Weight development history and predisposition to maintain weight or to gain weight appeared to be captured within the groups. Most participants in group one felt they had always maintained their weight throughout their life only putting on a few pounds at any time.

*“through my teens twenties I would have always maintained pretty much the same weight so if I gained weight I would be very aware of it”*

For group two some recalled always having weight on, for others weight crept up gradually over time.

*“I think I started putting on weight in my 20’s probably around 22/23. And it continued until I was eh 28”*

Weight gain for group two was attributed to getting married, slipping back into old habits and change of work.

*“It was very gradual. I didn’t really notice it. Kind of slipped back into old habits and it sort of crept on over a few years and then sort of escalated”*

In group three recollections of never being slim were recalled by most participants with experiences of either always having weight on, born a big baby or putting on in teens or by early adulthood. Some had gained and lost some weight several times and then put it back on later with extra weight added.

*“when I look back I would have been always have been a heavier baby and I was very big born I was you know over 10 pound born so I was the bigger baby”*

Weight gain later in life for group three was considered attributable to changing habits, getting married and no longer participating in sport. Participants advised getting married involved a more active social life or involved moving further away from family indicating that there were less supports available.

*“around when I was 28. Going out for a good while, got married when I was about 34, 33, that age group and I suppose around that stage we probably bought a house, we moved a bit further out of town”*

Lack of energy in the evening after a day’s work was also attributable to weight gain diminishing the ability to prepare healthier food and opting for quick fix meals.

#### Sub-theme: self-image

In group three being overweight for most participants brought with it lower self-esteem, self-loathing and feelings of disgust. In response to the question “So what would it mean to you then to be a healthy weight?” the response from one participant was as follows:

*“Oh god, it would mean everything. Absolutely everything, I can’t stand the sight of myself”*

Participants hated seeing photographs, looking in the mirror and having to shop for bigger more expensive clothes.

*“I mean I can’t bear going shopping for clothes. I tend to wear black most of the time. Em, not that I’d be particularly colourful anyway. But like I can’t see, you know I mean if it was my daughters Holy Communion and I was getting really worked up and upset about trying to find something to wear for that…”*

### Theme: cognitive dissonance

Participants in group one were efficient at taking action on what they knew to be favourable behaviours and actions. For most they described the ability to maintain their weight as a way of life.

*“So I get used to eating healthy and exercising as well so for me now it’s a way of living”.*

Group two reported that improved knowledge leads to a raised awareness and the ability to make better choices and do what you know you is right.

*“It’s finding your own way and having the confidence to find your own way. Some people don’t actually have the confidence to do what they think is right it’s facing up to what you think is right”*

While the desire to be fit, do more, have more energy and play with the children were noted by respondents in group three, turning knowledge into action was not prioritised in reality or in practice in their lives.

*“...... You know I want to be with you mammy where can we play mammy come on out here and play mammy on the floor mammy and mammy can’t even get on the floor in the first place”*

Participants were not unaware of the effects of their eating habits and exercise routines on their weight management as knowledge on portion size, calorie and nutrient content was high. Despite this, portion control was poor and binge eating often out of despondency was reported. This was acknowledged by the respondents themselves even though they seemed to be powerless in correcting it. Turning knowledge into action remained a difficulty for group three.

### Theme: role of employer

#### Sub-theme: negative versus positive perspectives

There were notable difference in attitudes to the role of their employer in facilitating healthy weight maintenance across the groups with those is group one and two reporting a campus that promoted healthy behaviours around food and physical activity. They quoted

*“Lots of options for exercise and choice of foods across campus”*

*“you can bring in own food and consume it on the premises”*

*“The University has a strong culture of sport and a strong emphasis on health”*

Participants in group three viewed the University more negatively in the way it facilitated healthy behaviours around food and physical activity and were more likely to blame the environment. They cited

*“Lack of availability of healthy foods”*

*“Lack of availability of exercise”*

*“Cost of healthy eating options on campus is prohibitive “*

### Theme : advice to others

Those in groups one and two were asked what advice they would give to those struggling with weight loss. The advice was mainly centered around four main areas, timely rowing back, maintaining balance between calories in and out, and establishing healthy eating and exercise as a way of life, as a routine. Patience and not being too hard on yourself also emerged as important. Acknowledge the need for treats and then row back. Watch calories even those from fruit and especially alcohol.

## Discussion

Obesity is a worldwide problem with dramatic impact on morbidity, mortality, quality of life and health care costs. The WHO now refers to the global pandemic of overweight and obesity as “globesity” [17]. The physical, financial and emotional outcomes of these diseases can be significant for the affected person and his/her family. In addition, the costs of these morbid conditions to society for treatment can be staggering as well, it has been previously estimated that the economic impact of obesity in Ireland is around €1.13 billion annually [6].

The findings emerging from this qualitative study illustrate the complex nature of healthy weight maintenance even amongst those who achieve it on an ongoing or lifelong basis. There are several important lessons to be learned from the primary weight maintainers included in this study, but these are not simple, and there is no one approach that can be applied to all. The nature of our society now requires a constant, cognitive effort to maintain a healthy weight, but this is not a passive process. Maintaining a healthy weight is no longer the “default consequence” of our lifestyles as it would have been some 30 years ago when the majority of the population walked/ cycled to school/work, and most had limited snacking options between meals and eating out was a treat that happened on special occasions once or twice a year. In those conditions in fact in would have been difficult for most individuals to become overweight or obese. To maintain a healthy weight now however people are required to take constant action in terms of planning, organisation, portion control, planned regular exercise, and to make these a priority in their lives. These findings appear to fit into the conceptual framework known as ‘positive deviance’. This framework has previously been used to study why some individuals achieve better weight loss outcomes than others exposed to the same resources and supports [18].

In addition will power, emotional wellbeing, resilience, good stress management, good social supports and even vanity, are all important. This highlights the interaction between and the interdependence of factors within and across all levels of a health problem [3]. Regulation, routine, balance, and individual timely recognition of weight gain, and rowing back, appear to be the cornerstone which prevents a spiral into positive energy balance and resultant weight gain. The idea of using the waist band as a marker or gauge, indicating that a rowing back is needed, is an interesting finding. As a public health message it is effective for primary weight maintainers, but it is also a simple message. In the clinical setting the waist band measurement is an important marker of central body obesity and the metabolic syndrome [19]. An important message that emerges from this study, and which may encourage those who struggle with their weight, is that lean people have to constantly strive to keep their weight in check too – that it does not just happen for them. This may bring some consolation to those who struggle.

Very few research papers have previously focused on primary weight maintainers but a recent study from Sweden [20] used a grounded theory approach to examine this in 23 primary weight maintainers. Primary weight maintainers who maintained their weight within healthy weight ranges described it as a “tightrope walk”. Maintaining balance could be easy or difficult depending on three factors: their pre-requisites for maintaining weight, their mental preparedness to maintain or change weight and the actions needed on their side to maintain weight. Interestingly very similar themes to ours emerged from their study including the need for self-control, establishing a routine, concerns around health and vanity, stress management, week day versus weekend behaviours and the value of good social supports. The Swedish paper concludes that public health advice given about weight, food habits and physical activity within the primary health care system needs to be nuanced and based on individual assessment or identification of subgroups of individuals with special needs. In our study we concur with these findings in that we highlighted balance, relaxing the rules at weekend but rowing back immediately after, sticking to a routine, advance organisation and planning around food and exercise, prioritisation, family, collegial and social supports, stress management and vanity, health and parental role modelling as motivating factors. This close corroboration of our results is all the more interesting considering the two different contexts in which the data was collected - Ireland versus Sweden and the fact that the study is a qualitative one and therefore you would not expect the results to be translatable to another population. A subsequent study by the Swedish group [21] suggested that healthy weight maintenance may be more complex for women than men due to care giving responsibilities and critical events such as childbearing. These themes also emerged in our data. Furthermore they suggest that interventions may even need to be tailored to the person’s demographic situation inclusive of age, sex, and body mass index. Again our data is also suggestive of this. We would go one further and suggest that life stage is also important as events like child bearing and transitionary periods such as starting a new job and events like relationship break up or divorce impacted on ability to maintain a healthy weight.

As a society we need to need to return to a situation where a healthy weight once again is a natural consequence of everyday living, because the effort currently required is not sustainable for the majority of the population. This will require joined-up thinking and multi-sectoral action between many agencies [7] in Ireland and far beyond, including the food industry, society and individuals. We need individuals to take responsibility for their own behaviours, within conditions that empower them to easily make healthy choices. Areas for exploration and innovation could include ways to encourage increased energy expenditure throughout the daily routine such as walking to school and creating an environment whereby supports are readily available and easily accessed by those who require them. Employer responsibilities to staff around food and exercise provision and health promotion may also be a route to explore. In some public sector settings such as the Irish Army there are programmes in place for early identification and referral pathways to the appropriate clinical staff for employees who stray out of normal BMI parameters (personal communication). Perhaps these models can be explored, and extended to other sectors of society. Early intervention and appropriate clinical staff to provide supports is an important consideration. Ready access to inexpensive professional advice may also be important, for example only one person in our group three had ever been to see a dietician, and cost seemed to be a prohibitive factor in this.

Recommendations for policy around primary prevention of weight gain may target lone families, particularly those with poor social supports, new mothers, and other subgroups at risk, in particular those in transitions in their lives such as new entrants to college/universities/work, those going through emotional upheaval such as marital breakup or grieving. There should be mandatory responsibilities for human resources in organisations to ensure that the correct conditions for health weight maintenance are in place, such as refrigeration, storage, preparation and cooking facilities for staff and students. Life coach and nutrition education should be part of mandatory training for all those in the workplace at work, and college. In addition professional expert exercise and nutrition counselling should be a service open to all employees at a reduced/minimal cost. Work place restaurants need to make the unhealthy options the opt out choice, remove unhealthy options to empower staff to make healthy options. Policies can’t make people make healthy choices but they can create the conditions that facilitate people to make good choices and conditions to promote and facilitate exercise. At an individual level it is noteworthy that logistical factors such as lack of organisation and planning were cited as deterrents to healthy choices. These factors should be relatively easily addressed by a life coach approach. The other individual factors such as willpower, and impulsivity may be more difficult issues to deal with but are not insurmountable either with the correct professional approach such as cognitive behavioural therapy by trained staff. In conclusion there is much to learn from this small study of primary weight maintenance both for individuals, society and policy makers. Further research is needed on larger samples to explore these issues.

### Limitations

The qualitative approach, the selectness of the population under study and the sample size are clear limitations of this study. As opposed to studies taking a phenomenological or grounded theory approach, where there is an expectation of a more in-depth interpretive analysis of findings, qualitative descriptive studies may be less interpretive, however, they are more in-depth than quantitative studies [16].

Also, it needs to be considered that due to self-reported weight development, participants may record their weight inaccurately, especially in female participants as it has been seen in previous research that women tend to under-report their weight [22]. It is encouraging however that the only other study which has explored primary weight maintenance which also utilized qualitative methods concurred with our findings despite being conducted in an entirely different context, Sweden [20].

## Conclusion

Our results shed light on the interplay of various factors related to healthy weight maintenance including personal perceptions, and personal motivations. We have also shown the importance of home, work and environmental conditions in encouraging participation in community and social activities, and supporting personal strategies that appear fundamental to carrying out successful goals and plans.

### Future work

Our first task is to disseminate the results of our study to relevant stakeholders within the health care system and beyond. Following on from this would like to develop and evaluate an intervention incorporating all of the elements identified as important to weight maintenance in this small study.

## Appendix A

### Topic guides

#### Focus group one. primary healthy weight maintainers group

Do you consciously maintain a healthy weight?

How do you do this?

How important is it to you to be a healthy weight?

Explore the following

Diet, Exercise, Alcohol, Strategies, Organisational issues/planning, Will power, Peer influences – home life, work colleagues, friends etc

What keeps you motivated?

Does work have any positive or negative impacts on this?

What could your employers do to help its employees to maintain/achieve a healthier weight?

Do you have strategies for rolling back on weight gain if you feel that you have put on a few pounds?

What advice would you give to others trying to achieve a healthy weight?

Any other comments?

#### Focus group two. Have achieved sustained weight loss

What age were you when you first put weight on?

Did the weight gain occur suddenly or was it slowly over time?

Was it associated with any particular event in your life?

What do you think was the cause of your weight gain?

How important is it to you to be a healthy weight ?

Did you know how to lose weight in theory but just could achieve it in reality?

Why do you think this was?

What lead to the change – in order word how did you get from that point to where you are now?

What motivated you to do this?

What behaviours did you change?

What else about you changed your life about?

What supports did you use?

Did you find these helpful?

Explore the following

Diet, Exercise, Alcohol

What advice would you give to others trying to achieve a healthy weight?

What could your employers do to help its employees to maintain/achieve a healthier weight?

#### Focus group three. Unsuccessful weight loss group

What age were you when you first put weight on?

Did the weight gain occur suddenly or was it slowly over time?

Was it associated with any particular event in your life?

How important is it to you to be a healthy weight ?

What do you think was the cause of your weight gain?

Diet – explore

Exercise -explore

Alcohol - explore

Strategies - explore

Organisational issues /planning – explore

Will power- explore

Peer influences – home life, work colleagues, friends etc

Does work have any positive or negative impacts on this?

What could your employers do to help facilitate your weight loss?

Do you know how to lose weight in theory but just can’t achieve it in reality?

Why do you think this is?

Since you first put on weight have you ever managed to lose weight even temporarily?

What motivated you to do this?

What caused you to slip back?

What supports have you used?

Did you find these helpful?

What do you think you would need to change in your life in order for you to be able to lose weight on a permanent basis?

Why can’t you implement these changes?

Any other comments?

## References

[1] World Health Organization: Obesity and Overweight Fact Sheet 2015 http://www.who.int/mediacentre/factsheets/fs311/en/

[2] Haslam DW, James WP (2005). Obesity. Lancet.

[3] Field AE, Coakley EH, Must A, Spadano JL, Laird N, Dietz WH (2001). Impact of overweight on the risk of developing common chronic diseases during a 10-year period. Arch Intern Med.

[4] Department of Children and Youth Affairs. Growing Up In Ireland Longitudinal Study of Children – The life of 9-year olds http://www.growingup.ie/fileadmin/user_upload/documents/Second_Child_Cohort_Reports/Growing_Up_in_Ireland_-_Overweight_and_Obesity_Among_9-Year-Olds.pdf

[5] The Over 50s In A Changing Ireland. Economic Circumstances, Health and Well-Being. The Irish Longitudinal Study on Ageing Health and Well-Being http://www.tcd.ie/tilda/publications/reports/w2-key-findings-report/Wave2-Key-Findings-Report.pdf

[6] The cost of overweight and obesity to the island of Ireland http://www.safefood.eu/SafeFood/media/SafeFoodLibrary/Documents/Publications/Research%20Reports/Final-Exec-Summary-The-Economic-Cost-of-Obesity.pdf

[7] DOHC The Report of the National Taskforce on Obesity. Obesity: the Policy Challenges http://lenus.ie/hse/bitstream/10147/46689/1/1744.pdf

[8] Written Answers - Departmental Reports http://debates.oireachtas.ie/dail/2011/07/12/00312.asp

[9] Kayman S, Bruvold W, Stern JS (1990). Maintenance and relapse after weight loss in women: behavioral aspects. American J Clin Nutr.

[10] IUNA National Adult Nutrition Survey – summary Report http://www.iuna.net/wp-content/uploads/2010/12/National-Adult-Nutrition-Survey-Summary-Report-March-2011.pdf

[11] Gatewood JG, Litchfield RE, Ryan SJ, Geadelmann JD, Pendergast JF, Ullom KK (2008). Perceived barriers to community-based health promotion program participation. Am J Health Behav.

[12] Elfhag K, Rossner S (2005). Who succeeds in maintaining weight loss? A conceptual review of factors associated with weight loss maintenance and weight regain. Obes Rev.

[13] Wing RR, Hill JO (2001). Successful weight loss maintenance. Annu Rev Nutr.

[14] Proctor S, Allan T, Lacey A, Gerrish K, Lacey A (2010). The research process in nursing. Sampling.

[15] Boyatzis RE (1998). Transforming qualitative information: thematic analysis and code development.

[16] Sandelowski M (2000). Focus on research methods whatever happened to qualitative description?. Res Nurs Health.

[17] World Health Organization Controlling the global obesity epidemic. 2015. http://www.who.int/nutrition/topics/obesity/en/

[18] Stuckley HL, Boan J, Kraschnewski JL, Miller-Day M, Lehman EB, Sciamanna CN: Using positive deviance for determining successful weightcontrol practices. Qualitative Health Research. 2011;21:563-579. 10.1177/1049732310386623 PMC3612888 20956609

[19] Safefood.eu: Stop the spread campaign. http://www.safefood.eu/SafeFood/media/SafeFoodLibrary/Documents/Professional/Events/Marian-Faughnan.pdf

[20] Lindvall K, Larsson C, Weinehall L, Emmelin M: Weight maintenance as a tight rope walk – a Grounded Theory study. BMC Public Health. 2010;10:51. 10.1186/1471-2458-10-51 PMC2835685 20122140

[21] Lindvall K, Jenkins P, Emmelin M, Scribani M, Norberg M, Larsson C, Weinehall L: Primary weight maintenance: an observational study exploring candidate variables for intervention. Nutrition Journal. 2013;12:97. 10.1186/1475-2891-12-97 PMC3717287 23855935

[22] Nawaz H, Chan W, Abdulrahman M, Larson D, Katz DL: Self-reported weight and height: implications for obesity research. American Journal of Preventative Medicine. 2001;297:298. 10.1016/s0749-3797(01)00293-8 11331120

[23] World Health Organization Controlling the global obesity epidemic 2015 http://www.who.int/nutrition/topics/obesity/en/

